# Platelet abnormalities in patients with Parkinson's disease undergoing preoperative evaluation for deep brain stimulation

**DOI:** 10.1038/s41598-022-18992-1

**Published:** 2022-08-26

**Authors:** Sheng-Che Chou, Chun-Hwei Tai, Sheng-Hong Tseng

**Affiliations:** 1 Department of Traumatology, National Taiwan University Hospital, No. 7, Chung Shan S. Rd., Taipei, 100225 Taiwan; 2 Department of Neurology, National Taiwan University Hospital, Taipei, Taiwan; 3 Division of Neurosurgery, Department of Surgery, National Taiwan University Hospital, Taipei, Taiwan; 4 Graduate Institute of Clinical Medicine, College of Medicine, National Taiwan University, Taipei, Taiwan

**Keywords:** Parkinson's disease, Parkinson's disease, Parkinson's disease

## Abstract

Normal hemostatic function is important for reduction of the risk of intracranial hemorrhage during stereotactic neurosurgery including deep brain stimulation (DBS) surgery. This study investigates the hemostatic function in patients with Parkinson’s disease (PD) undergoing preoperative evaluation for DBS, with emphasis on the number and function of platelets. In 107 PD patients, only one had abnormal activated partial prothrombin time and normal prothrombin time. Among the other 106 patients, six (5.7%) had only thrombocytopenia, seven (6.6%) only prolonged bleeding time (BT), and 14 (13.2%) only prolonged closure time (CT) of platelet function analyzer 100 (PFA-100). Totally, 34 of the 106 patients (32.1%) had at least one of three kinds of platelet abnormalities. No factor was found to be associated with the occurrence of platelet abnormalities except that abnormal platelet group and prolonged BT subgroup had more patients using selegiline and lower UPDRS-III motor subscore with medication off than normal platelet group (*p* < 0.05). The use of selegiline was significantly correlated with prolonged BT (*p* = 0.0041) and platelet abnormality (*p* = 0.0197). Therefore, it is important to have detailed evaluation of the hemostatic function for PD patients undergoing preoperative evaluation for DBS, especially the platelet number and function.

## Introduction

Parkinson’s disease (PD) is a degenerative disorder of the central nervous system that presents impairment of motor functions. In recent years, deep brain stimulation (DBS) has become a widely accepted treatment method for PD patients^1^. The DBS surgery, a kind of stereotactic surgery, always harbors the risk of intracranial hemorrhage (ICH) during surgery^2–4^. In DBS surgery, DBS electrodes are implanted to the targets deep in the brain and intraoperative microelectrode recording is often adopted to identify the locations of the targets. All these procedures need repeated insertion of the equipment into the brain, and carry the risk of vascular injury and ICH. Although the risk of ICH can be minimized by well-controlled blood pressure, meticulous surgical techniques, and careful trajectory planning, the incidence of ICH caused by DBS surgery has been found to range from 0.8 to 5.3%^2–8^.

Hemostasis for the ICH that is bleeding during DBS surgery mainly depends on the clotting and coagulation functions of patients. Therefore, preoperative evaluation of hemostatic function is mandatory and any abnormalities of hemostatic function should be corrected either before or during the surgery. In the literature, coagulopathy^9^, thrombocytopenia^10–12^, or platelets dysfunction^13^ in PD patients had been mentioned in several reports. However, it is still unclear whether the PD patients have hemostatic dysfunction, especially the patients having received medical treatment for more than 5 years and being considered for candidate of DBS surgery. This article investigates the hemostatic function in patients with PD undergoing preoperative evaluation for DBS, with emphasis on the number and function of platelets.

## Methods

The PD patients undergoing preoperative evaluation for DBS surgery from January 2015 to December 2017 were investigated. This study was approved by the Research Ethics Committee of National Taiwan University Hospital (201909010RIND) and all methods were carried out in accordance with relevant guidelines and regulations. Patients with the diagnosis of PD for more than five years and with at least one of the following conditions-marked motor fluctuation, severe levodopa-induced dyskinesia, and intractable tremor-were included for preoperative evaluation. Patients with diagnosis of atypical parkinsonism, poor response to levodopa treatment, dementia, depression, psychiatric symptoms at low dosage of levodopa treatment, or systemic illness that influenced the perioperative safety of the patient were excluded from evaluation. In addition, patients having hematological disorder, renal insufficiency, liver cirrhosis, being treated with antiplatelet or anticoagulation agents, or nonsteroidal anti-inflammatory drugs were excluded from this study.

The age, sex, duration of symptoms, Hoehn and Yahr stage with medication off and medication on, Unified Parkinson’s Disease Rating Scale (UPDRS)-III motor subscore with medication off and medication on, PD medications, levodopa dosage, levodopa equivalent daily dose (LEDD), prothrombin time (PT), activated partial prothrombin time (aPTT), platelet count, closure time (CT) of Platelet Function Analyzer 100 (PFA-100), and bleeding time (BT) of the PD patients were retrospectively reviewed and analyzed. The normal range of PT is 9.8–11.5 s, aPTT 25.5–32.6 s, platelets counts 150,000–361,000/μl. The CT of PFA-100 was measured using PFA-100® system (Siemens, Germany). The normal range of CT of PFA-100 is 91–175 s for collagen/epinephrine (Col/EPI) test, and 61–109 s for collagen/adenosine phosphate (Col/ADP) test, respectively. Abnormal value of either Col/EPI or Col/ADP test was defined as abnormal CT of PFA-100. The bleeding time was measured using Surgicutt® (Accriva Diagnostics, USA) and the normal range is 2–8 min.

The difference of the age, sex, duration of symptoms, Hoehn and Yahr stage and UPDRS-III motor subscore during medication off and on periods, PD medications, levodopa dosage, and LEDD between the normal and thrombocytopenia, prolonged BT, prolonged CT of PFA-100 or abnormal platelet groups was analyzed by Chi-square tests or Fisher's exact tests for dichotomous variables, and independent-samples t-tests for continuous variables. In the univariate logistic regression analysis, comparisons between each group’s variables were made. Multivariate analysis was conducted by fitting a logistic regression model to identify risk factors of platelet abnormalities. The statistical analysis was performed with Statistical Analysis Software (version 9.4). The statistical significance was accepted as *p* < 0.05.

### Ethics declarations

This study was approved by the Research Ethics Committee of National Taiwan University Hospital (201909010RIND).

### Consent to participate

Informed consent was not needed from all patients included in the study according to the approval of Research Ethics Committee of National Taiwan University Hospital.

## Results

During the study period, there were 112 patients with PD undergoing preoperative evaluation for DBS surgery. Five patients treated with antiplatelet or anticoagulant agents were excluded from this study. In the remaining 107 patients, one (0.9%) patient had abnormal aPTT (37.6 s) and normal PT; however, he had prolonged BT (8.5 min) and normal platelet count and CT of PFA-100. The other 106 patients (99.1%) had normal PT and aPTT, and they were subjected to the analysis of platelet abnormality, excluding the patient with abnormal aPTT.

Table 1 shows the demographic data of the 106 patients with Parkinson’s disease. Among these patients, 51 (48.1%) were females and 55 (51.9%) were males. The age ranged from 35 to 77 (mean ± standard deviation, 62.5 ± 7.4) years old. The duration of symptoms in these patients ranged from 5 to 37 (11.7 ± 5.4) years. The Hoehn and Yahr stage ranged from stage 2 to 5 (3.6 ± 0.6) with medication off, and stage 1 to 5 (2.5 ± 0.6) with medication on. The UPDRS-III motor subscore ranged from 16 to 95 (41.4 ± 12.5) with medication off, and 3 to 40 (18.7 ± 8.7) with medication on. All 106 patients had normal PT (10.3 ± 0.5 s) and aPTT (27.6 ± 2.0 s). PD medications received by these patients included levodopa/benserazide, entacapone, amantadine, carbidoma/levodopa, pramipexole, ropinirole, biperiden, rotigotine, selegiline, trihexyphenidyl, and azilect. The levodopa dosage ranged from 150 to 2400 mg/day (865.7 ± 383.8 mg/day). The LEDD ranged from 165 to 2805 mg/day (1341.4 ± 501.0 mg/day).

**Table 1:** Demography of patients with Parkinson’s disease.

Characteristics	PD patients (n = 106)
Age (years)	35–77 (62.5 ± 7.4)
Sex (Female : Male)	51:55
Duration of symptoms (years)	5–37 (11.7 ± 5.4)
**Hoehn and Yahr stage**	
Off	2–5 (3.6 ± 0.6)
On	1–5 (2.5 ± 0.7)
**UPDRS-III**	
Off	16–95 (41.4 ± 12.5)
On	3–40 (18.7 ± 8.7)
PT (second)	9.3–11.5 (10.3 ± 0.5)
aPTT (second)	22.2–32.6 (27.6 ± 2.0)
Levodopa (mg/day)	150–2400 (865.7 ± 383.8)
LEDD (mg/day)	165–2805 (1341.4 ± 501.0)
**PD medications**	
Levodopa/benserazide	74 (69.8%)
Entacapone	56 (52.8%)
Amantadine	56 (52.8%)
Carbidoma/levodopa	48 (45.3%)
Pramipexole	45 (42.5%)
Ropinirole	32 (30.2%)
Biperiden	23 (21.7%)
Rotigotine	14 (13.2%)
Selegiline	9 (8.5%)

PD, Parkinson’s disease; UPDRS, Unified Parkinson’s Disease Rating Scale; PT, prothrombin time; aPTT, activated partial prothrombin time; LEDD, levodopa equivalent daily dose.

**Figure 1:**
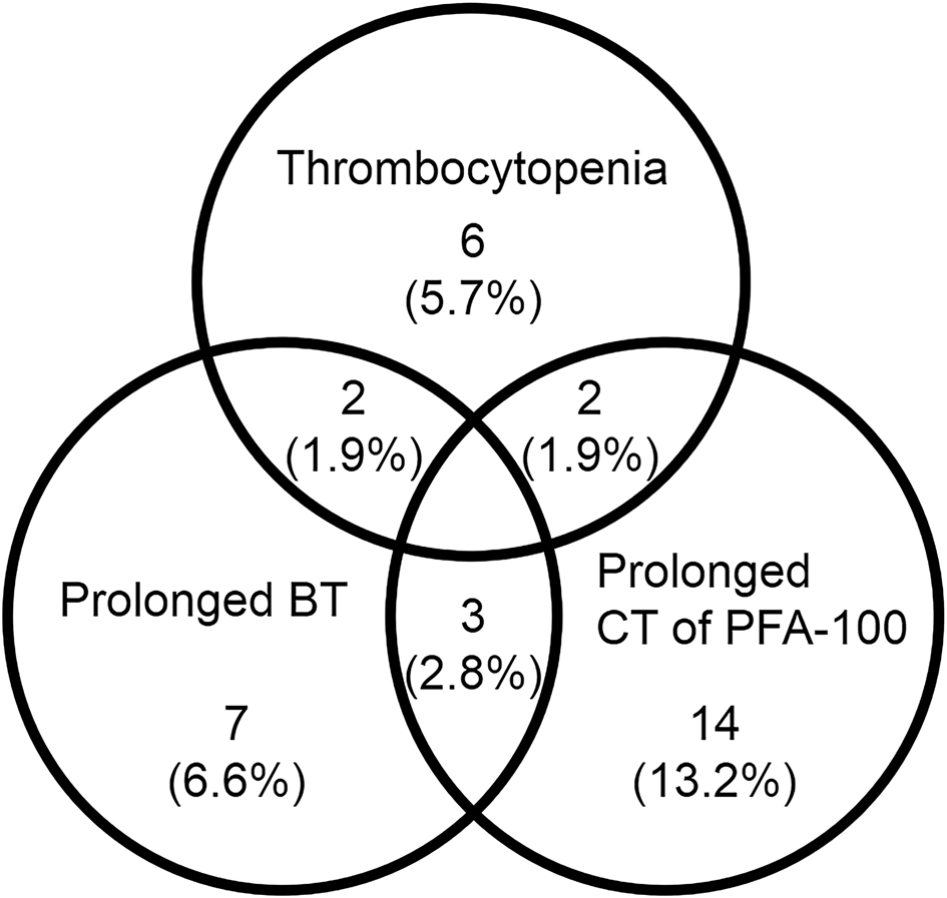
The number and percentage of patients with thrombocytopenia, prolonged closure time of platelet function analyzer 100 (CT of PFA-100), and/or prolonged bleeding time (BT) in 106 patients with Parkinson’s disease.

Figure 1 shows the number and percentage of patients having thrombocytopenia, abnormal CT of PFA-100 and/or prolonged bleeding time in these 106 PD patients. Totally 34 patients (32.1%) had at least one of these three kinds of platelet abnormalities. Ten patients (9.4%) had thrombocytopenia, 12 (11.3%) prolonged BT, and 19 (17.9%) prolonged CT of PFA-100. In these patients, six (5.7%) had only thrombocytopenia, seven (6.6%) only prolonged BT, and 14 (13.2%) only prolonged CT of PFA-100. Two patients (1.9%) had both thrombocytopenia and prolonged BT, two (1.9%) had both thrombocytopenia and prolonged CT of PFA-100, and three (2.8%) had both prolonged BT and prolonged CT of PFA-100.

The 106 patients can be divided into two groups: normal platelet (72 patients, 67.9%) and abnormal platelet (34 patients, 32.1%) groups. The abnormal platelet group was further divided into 3 subgroups: thrombocytopenia (10 patients, 9.4%), prolonged BT (12 patients, 11.3%), and prolonged CT of PFA-100 (19 patients, 17.9%). The difference of age, sex, duration of symptoms, Hoehn and Yahr stage, UPDRS-III motor subscore, levodopa dosage, LEDD, and PD medication between normal platelet and abnormal platelet or each subgroup were analyzed (Table 2). There was no difference in the age, sex, Hoehn & Yahr staging, UPDRS-III motor subscore with medication on, the dose of levodopa, and LEDD between these two groups (*p* > 0.05), except that the UPDRS-III motor subscore with medication off was higher in the normal platelet group than in the abnormal platelet group (*p* = 0.0418) and prolonged BT subgroup (*p* = 0.0249). There was no difference in the PD drugs used by the patients between these two groups, except that the abnormal platelet group had more patients using selegiline than the normal platelet group (*p* = 0.0290), and the prolonged BT subgroup also had more patients using selegiline than the normal platelet group (*p* = 0.0070). In the univariate logistic regression analysis (Table 3), we found that use of selegiline was significantly associated with prolonged BT (*p* = 0.0041, OR 11.500) and abnormal platelet (*p* = 0.0315, OR 4.929). The UPDRS-III motor subscore with medication off was also significantly associated with abnormal platelet (*p* = 0.0464, OR 0.960). In the multivariate analysis (Table 4), lower UPDRS-III motor subscore with medication off (*p* = 0.0316, OR 0.954) and use of selegiline (*p* = 0.0197, OR 6.069) were significantly associated with platelet abnormality when the other variables were controlled.

**Table 2:** Comparison of various factors in PD patients between normal platelet and abnormal platelet group or each test.

Characteristics	Normal platelet (n = 72)	Abnormal platelet (n = 34)	*p* value	Thrombocytopenia (n = 10)	*p* value	Prolonged BT (n = 12)	*p* value	Prolonged CT of PFA-100 (n = 19)	*p* value
Age (years)	35–74 (62.7 ± 7.6)	47–77 (62.1 ± 7.1)	0.7239	56–73 (65.3 ± 6.2)	0.3008	48–77 (63.2 ± 8.0)	0.8429	47–73 (61.6 ± 6.8)	0.5610
Sex (Female : Male)	35:37	16:18	0.881314	2:8	0.1046	6:6	0.9290	10:9	0.7552
Duration of symptoms (years)	5–37 (12.3 ± 5.4)	5–28 (10.5 ± 5.3)	0.1122	5–15 (9.5 ± 3.8)	0.1143	6–28 (12.6 ± 6.9)	0.8806	6–24 (10.3 ± 5.2)	0.1496
**Hoehn and Yahr stage**									
Off	2–5 (3.7 ± 0.6)	3–4 (3.5 ± 0.5)	0.2076	3–4 (3.5 ± 0.5)	0.3698	3–4 (3.5 ± 0.5)	0.3302	3–4 (3.6 ± 0.5)	0.5012
On	1–5 (2.5 ± 0.7)	1–3 (2.3 ± 0.6)	0.0732	2–3 (2.4 ± 0.5)	0.5348	2–3 (2.3 ± 0.5)	0.3198	1–3 (2.3 ± 0.7)	0.2056
**UPDRS-III**									
Off	22–95 (43.1 ± 13.0)	16–60 (37.8 ± 10.6)	0.0418*	22–58 (40.6 ± 11.2)	0.5641	25–51 (36.9 ± 7.2)	0.0249*	16–60 (38.9 ± 12.0)	0.2064
On	3–40 (19.7 ± 8.5)	5–40 (16.7 ± 8.8)	0.1048	7–32 (19.1 ± 9.0)	0.8413	6–40 (24.4 ± 9.1)	0.7840	5–40 (16.7 ± 9.8)	0.1898
Levodopa (mg/day)	200–1900 (858.9 ± 334.7)	150–2400 (880.1 ± 477.0)	0.7789	350–2400 (1040.0 ± 574.4)	0.3422	150–1500 |(806.3 ± 412.1)	0.6601	150–1750 (842.1 ± 447.0)	0.8957
LEDD (mg/day)	350–2645 (1315.3 ± 445.8)	165–2805 (1396.5 ± 605.3)	0.4871	625–2550 (1447.7 ± 651.5)	0.4088	165–2420 (1327.1 ± 635.0)	0.9357	165–2805 (1378.1 ± 637.2)	0.6890
**PD medications**									
Levodopa/benserazide	52 (72.2%)	22 (64.7%)	0.4314	7 (70.0%)	1.0000	7 (58.3%)	0.3292	13 (68.4%)	0.7442
Amantadine	38 (52.8%)	18 (52.9%)	0.9874	4 (40.0%)	0.5142	6 (50.0%)	0.8584	10 (52.6%)	0.9909
Entacapone	38 (52.8%)	18 (52.9%)	0.9874	4 (40.0%)	0.5142	8 (66.7%)	0.3708	10 (52.6%)	0.9909
Carbidoma/levodopa	29 (40.3%)	19 (55.9%)	0.2206	4 (40.0%)	1.0000	7 (58.3%)	0.2419	10 (52.6%)	0.3331
Pramipexole	28 (38.9%)	17 (50.0%)	0.4256	5 (50.0%)	0.5134	4 (33.3)	1.0000	9 (47.4%)	0.5033
Ropinirole	21 (29.2%)	11 (32.4%)	0.7387	4 (40.0%)	0.4840	5 (41.7)	0.5014	5 (26.3%)	0.8067
Biperiden	19 (26.4%)	4 (11.8%)	0.0882	1 (10.0%)	0.4376	1 (8.3)	0.2776	4 (21.1%)	0.7715
Rotigotine	11 (15.3%)	3 (8.8%)	0.5407	0 (0.0%)	0.3435	2 (0.17)	1.0000	2 (10.5%)	0.7290
Selegiline	5 (6.9%)	4 (11.8%)	0.0290*	0 (0.0%)	1.0000	4 (33.3)	0.0070*	3 (15.8%)	0.1029

PD, Parkinson’s disease; BT, bleeding time; CT, closure time; PFA-100, Platelet Function Analyzer-100; UPDRS, Unified Parkinson’s Disease Rating Scale; LEDD, levodopa equivalent daily dose.

*Statistically significant.

**Table 3:** Univariate analysis of the factors affecting platelet count and function in PD patients.

Variables	Thrombocytopenia				Prolonged BT				Prolonged CT of PFA-100				Abnormal platelet		
	OR	95% CI	*p* value		OR	95% CI	*p* value		OR	95% CI	*p* value		OR	95% CI	*p* value
Age	1.058	0.952, 1.175	0.2974		1.009	0.928, 1.096	0.8406		0.980	0.917, 1.048	0.5570		0.990	0.937, 1.046	0.7211
Sex	0.264	0.052, 1.332	0.1068		1.057	0.311, 3.589	0.9290		1.175	0.427, 3.232	0.7553		0.940	0.415, 2.127	0.8813
Duration of symptoms	0.858	0.712, 1.033	0.1064		1.008	0.906, 1.123	0.8788		0.915	0.810, 1.033	0.1508		0.930	0.850, 1.018	0.1170
**Hoehn and Yahr stage**															
Off	0.606	0.204, 1.798	0.3669		0.603	0.219, 1.661	0.3278		0.742	0.314, 1.755	0.4969		0.631	0.308, 1.291	0.2072
On	0.724	0.265, 1.981	0.5293		0.615	0.238, 1.590	0.3153		0.613	0.288,1.305	0.2044		0.554	0.288, 1.064	0.0760
**UPDRS-III**															
Off	0.983	0.927, 1.042	0.5595		0.943	0.877, 1.014	0.1135		0.970	0.926, 1.017	0.2068		0.960	0.923, 0.999	0.0464*
On	0.992	0.917, 1.073	0.8390		1.010	0.941, 1.085	0.7810		0.960	0.902, 1.021	0.1902		0.960	0.913, 1.009	0.1069
Levodopa dosage	1.001	1.000, 1.003	0.1539		1.000	0.998, 1.001	0.6559		1.000	0.998, 1.001	0.8943		1.000	0.999, 1.001	0.7485
LEDD	1.001	0.999, 1.002	0.4051		1.000	0.999, 1.001	0.9347		1.000	0.999, 1.001	0.6157		1.000	1.000, 1.001	0.4339
**PD medications**															
Levodopa/benserazide	0.897	0.211, 3.816	0.8835		0.538	0.153, 1.895	0.3349		0.833	0.278, 2.494	0.7444		0.705	0.295, 1.687	0.4324
Amantadine	0.596	0.155, 2.294	0.4522		0.895	0.263, 3.038	0.8585		0.994	0.361, 2.736	0.9909		1.007	0.445, 2.279	0.9875
Entacapone	0.596	0.155, 2.294	0.4522		1.789	0.494, 6.477	0.3753		0.994	0.361, 2.736	0.9909		1.007	0.445, 2.279	0.9875
Carbidoma/levodopa	0.989	0.256, 3.813	0.9866		2.076	0.600, 7.177	0.2485		1.648	0.596, 4.552	0.3356		1.668	0.733, 3.794	0.2223
Pramipexole	1.572	0.417, 5.925	0.5043		0.786	0.216, 2.855	0.7142		1.414	0.511, 3.913	0.5043		1.397	0.613, 3.182	0.4263
Ropinirole	1.619	0.414, 6.330	0.4885		1.735	0.494, 6.086	0.3897		0.867	0.277, 2.714	0.8068		1.161	0.482, 2.800	0.7388
Biperiden	0.310	0.037, 2.612	0.2814		0.254	0.031, 2.098	0.2032		0.744	0.219, 2.523	0.6349		0.372	0.116, 1.195	0.0968
Rotigotine	0.000	NA	0.9587		1.109	0.213, 5.766	0.9020		0.652	0.132, 3.230	0.6008		0.537	0.139, 2.066	0.3654
Selegiline	0.000	NA	0.9787		11.500	2.173, 60.865	0.0041*		4.313	0.796, 23.376	0.0901		4.929	1.152, 21.091	0.0315*

PD, Parkinson’s disease; BT, bleeding time; CT, closure time; PFA-100, Platelet Function Analyzer-100; OR, odds ratio; CI, confidence interval; UPDRS, Unified Parkinson’s Disease Rating Scale; LEDD, levodopa equivalent daily dose.

*Statistically significant.

**Table 4:** Multivariate analysis of the factors between normal and abnormal platelet groups in PD patients.

Variable	Estimate	Standard error	Wald Chi-square	*p* value	Odds ratio	95% Confidence interval
Intercept	0.9751	0.8730	1.2476	0.2640	–	–
UPDRS-III off	− 0.0473	0.0220	4.6203	0.0316*	0.954	0.914, 0.996
Selegiline	1.8032	0.7733	5.4377	0.0197*	6.069	1.333, 27.625

PD, Parkinson’s disease; UPDRS, Unified Parkinson’s Disease Rating Scale.

*Statistically significant. (*p* < 0.05).

## Discussion

The hemostatic function depends on both coagulation and clotting systems. The association between PD and coagulation abnormalities had been noted previously^9^. A study found that the 160 patients taking PD medications had higher average values of PT and plasma levels of prothrombin fragment_1+2_, D-dimer, plasmin-α2 antiplasmin complex, thrombomodulin and E-selectin than the 110 patients without any medication or the 159 healthy controls^9^. In addition, PD patients receiving combination therapy of levodopa and dopamine agonist have higher values of these hemostatic markers than those treated with only levodopa or dopamine agonist^9^. Furthermore, abnormalities in coagulation functions are more prominent in patients with more severe disease conditions (higher Hoehn and Yahr stage) and longer histories of illness and medical therapy^9^. These results suggest the coagulation abnormalities in PD patients are related to the duration and severity of PD, treatment with antiparkinsonian agents, multiplicity of PD drugs, and duration of treatment^9^. However, our study found that 106 of the 107 PD patients (99.1%) had normal coagulation functions and only one patient (0.9%) had prolonged aPTT, which suggests that most PD patients had normal coagulation function. In previous study^9^, the age of the patients treated with antiparkinsonian agents was 59.7 ± 14.0 years old, the duration of disease/therapy duration was 4.8 ± 2.2/4.0 ± 5.4 years, and the Hoehn and Yahr stage was 3.1 ± 0.9. However, the age of our patients was 62.5 ± 7.4 years old, the duration of disease was 11.7 ± 5.4 years, and the Hoehn and Yahr stage at medication off condition was 3.6 ± 0.6. These data, being similar to or higher than those in previous study^9^, do not support the viewpoints that patients with more severe disease, longer histories of illness and medical therapy are associated with coagulation abnormalities^9^. The inconsistency between our results and the previous report^9^ is unclear and deserves further investigation. Although the prevalence of abnormal PT or aPTT (0.9%) is very low in our study, it is still necessary to have PT/aPTTT test in preoperative evaluation, because they can be affected by medical disease or medication other than PD drugs.

In addition to the coagulation system, the clotting system, especially the quantity and quality of platelets, is also the major component of hemostatic function. In this study, we examined the platelet number and function, and found that 32.1% (34/106) of PD patients had at least one of three kinds of platelet abnormalities (thrombocytopenia, prolonged BT and/or prolonged CT of PFA-100). Platelets have been found to have similar structural, functional and biochemical characteristics to the neurons in a variety of neurodegenerative disorders (NDDs) including PD^14^. The neuronal loss in NDDs is linked to mitochondrial damage, and platelets under pathological conditions or physiological stimulation also show mitochondrial damage, in which platelets undergo anaerobic glycolysis and oxidative phosphorylation, and release reactive oxygen species to induce oxidative injury^14–16^. Such phenomenon is further revealed by experiments in the neural toxin 1-methyl-4-phenyl-1,2,3,6-tetrahydropyridine (MPTP) in animals^17^. The neuronal accumulation of 1-methyl-4-phenylpyridinium ion (MPP^+^), the metabolite of MPTP, can induce PD in humans^17^. In addition, MPP^+^ can impair the energy metabolism, cause the loss of intracellular ATP stores, and decrease the ATP secretion in both neurons and platelets^17^. MPP^+^ also decreases platelet aggregation activity in PD patients^17^. These findings suggest that PD itself or any insults that induce PD may result in platelet dysfunction^17^.

In PD patients, platelet count has been found to be usually in the normal range and only sporadic case reports of thrombocytopenia had been reported^10–13,18,19^. Our study revealed that 9.4% of PD patients had thrombocytopenia, which suggests the incidence of thrombocytopenia might not be as low as reported in the literature. The development of thrombocytopenia has been considered to be related to the levodopa treatment, especially after long-term levodopa treatment, and the platelet count usually recovers after withdrawal of levodopa^10–12,19^. In addition, thrombocytopenia in PD patients may also be the result of autoimmune reaction-induced platelet destruction, because these patients often present with positive antiplatelet, antinuclear or anti-erythrocyte autoantibodies^11,12,19^. Patients taking levodopa for three months or more had 8.8% to 9.5% incidence of positive reaction for Coombs test and 11.3% incidence of positive reaction for antinuclear antibody test^19,20^. The autoimmune reaction-induced thrombocytopenia is not dependent on the presence of levodopa because serologic abnormalities persist longer after levodopa is withdrawn^12^. In addition, the thrombocytopenia in a patient did not recur when purified levodopa was used to treat PD symptom, which indicating the thrombocytopenia is not due to direct allergy to levodopa in this patient^12^. Our patients did not receive immunological examination, thus, we have no comments about the autoimmune reaction.

Platelet function is also important for the hemostasis^17^. The data in the literature in PD patients are variable. A study reported that 10 PD patients had no change of bleeding time and platelet aggregation with ADP, epinephrine, and collagen at different dilution^18^. However, in another study about 25 PD patients, none had thrombocytopenia, but the platelet aggregation induced by ADP and epinephrine was significantly decreased (32% and 60%, respectively), while collagen-induced aggregation was unchanged, as compared with 25 control subjects^13^. In this report, we used two methods (BT and CT of PFA-100) to investigate the platelet function and found that 28 patients (26.4%) showed abnormality in either BT test or CT of PFA-100. PFA-100 is one of the clinically available tests based on platelet adhesion under shear stress^21–23^. Isolated prolonged Col/EPI closure time is possibly due to use of acetylsalicylic acid, or platelet dysfunction^23,24^. Both prolonged Col/EPI and Col/ADP CTs are possibly due to von Willebrand disease, Glanzmann thrombasthenia, Bernard-Soulier syndrome, Grey platelet syndrome, or other diseases^23,24^. Some other conditions, such as bone marrow disorders, congenital or acquired platelet disorders, and drug-induced platelet disorders, can also contribute to prolonged CT of PFA-100^22,25^. Our patients showed no such diseases or conditions mentioned above, and we found 17.9% patients had prolonged CT of PFA-100. In addition to PFA-100 test, BT test using Ivy or Duke Method has long been used for evaluating the platelet function, but the results of BT can be confounded by many factors, such as age, sex, direction of incision, vigorous exercise, variations in cuff pressure, excessive wiping the incision, excessive anxiety, and cold applied to adjacent skin^26^. Therefore, this test has been suggested not to be adopted as a routine preoperative test for patients without history of bleeding disorder by the College of American Pathologists and American Society of Clinical Pathologists since 1998, because a normal bleeding time cannot exclude excessive hemorrhage during surgery^26^. However, we think this test mimics the response to vascular injury caused by the insertion of microelectrodes or macroelectrodes during stereotactic surgery. Furthermore, although normal bleeding time cannot exclude the risk of intraoperative hemorrhage, abnormal BT could remind us the patient’s platelet function is abnormal. Therefore, we still included BT as one of the preoperative tests to evaluate the platelet function in our patients, and we found 12 patients (11.3%) had prolonged BT. For these two platelet function tests (BT and CT of PFA-100), our results revealed nine patients (8.5%) had only prolonged BT, 16 (15.1%) had only prolonged CT of PFA-100, and only three patients (2.8%) showed abnormality in both bleeding time and CT of PFA-100 tests. Therefore, we thought these two tests are complementary in detecting the abnormal platelet function.

The causes of the platelet abnormality in PD patients are intriguing, and have been ascribed to PD itself or PD medications^10–12,17,19^. In our study, we found the development of platelet abnormalities was not related to the age, sex, duration of symptoms, Hoehn and Yahr stage and UPDRS-III motor subscore during on periods. However, the normal platelet group had higher UPDRS-III motor subscore than the abnormal platelet group and the prolonged BT subgroup, which suggests the severity of PD is not correlated with the development of platelet abnormalities. PD medications may also induce platelet abnormalities. As mentioned above, levodopa has been reported to be associated with thrombocytopenia^10–12^; however, the dosage of levodopa was not significantly correlated with the platelet abnormalities in our study. In contrast, we found that the abnormal platelet group had more patients using selegiline than the normal platelet group, and the use of selegiline was significantly correlated with prolonged bleeding time. Selegiline is a selective monoamine oxidase (MAO) B inhibitor. The amount of platelet MAO activity is directly correlated with epinephrine-induced platelet aggregation, and the suppression of MAO activity by selegiline may thus affect the platelet function and bleeding time^27^. Nevertheless, selegiline was not significantly associated with prolonged CT of PFA-100 in our patients. More studies are necessary to clarify the effect of selegiline on platelet function.

Bleeding after stereotactic biopsy has a significant correlation only with the platelet count less than 150,000/mm^3^ (*p* = 0.006)^28^. Bleeding time is an in vivo test for hemostasis and can be considered as the simulation of bleeding during surgery. PFA-100 screening before DBS surgery and the administration of tranexamic acid to patients with prolonged CT appeared to lower the risk of an ICH by 1.8% when compared with patients without PFA-100 screening^29^. Therefore, the bleeding risk of patients undergoing stereotactic neurosurgery is increased when these tests are abnormal, and management should be taken before surgery. Among the 106 patients included in this study, 95 patients (89.6%) underwent DBS surgery after preoperative evaluation and 30 patients of them (31.6%) had at least one of three kinds of platelet abnormalities. Platelet transfusion was performed in patients with platelet abnormality before or during the surgery, and there was no symptomatic intracranial hemorrhage.

This study investigated PD patients undergoing preoperative evaluation for DBS surgery and selection bias might have occurred, because patients with early or late stage PD might not be included in this study population. Further study and larger study populations are necessary to clarify the relationship between platelet abnormalities and PD.

In conclusion, this study revealed that about one third of PD patients were associated with thrombocytopenia and/or platelet dysfunction, which suggests platelet abnormalities may occur in PD patients even though the patients do not have history of hematological disorders, taking antiplatelet or anticoagulation drugs, or other diseases that may impair the clotting or coagulation functions. Therefore, it is important to have a detailed preoperative evaluation of the platelet number and function before DBS surgery for PD patients. Our study showed that CT of PFA-100 had the highest sensitivity to reveal the platelet abnormality among the three parameters.

However, not all the platelet abnormalities in PD patients could be identified by any single test, thus we suggest the evaluation should include as much tests as the hospital can provide to increase the detection rate of platelet abnormalities. Whenever there are platelet abnormalities, either the surgery should be postponed or some management such as platelet transfusion should be performed before or during the surgery.

## Data Availability

The datasets generated during and analyzed during the current study are available from the corresponding author on reasonable request.
