# Publisher Correction: Platelet abnormalities in patients with Parkinson's disease undergoing preoperative evaluation for deep brain stimulation

**DOI:** 10.1038/s41598-022-21123-5

**Published:** 2022-10-03

**Authors:** Sheng-Che Chou, Chun-Hwei Tai, Sheng-Hong Tseng

**Affiliations:** 1 Department of Traumatology, National Taiwan University Hospital, No. 7, Chung Shan S. Rd., Taipei, 100225 Taiwan; 2 Department of Neurology, National Taiwan University Hospital, Taipei, Taiwan; 3 Division of Neurosurgery, Department of Surgery, National Taiwan University Hospital, Taipei, Taiwan; 4 Graduate Institute of Clinical Medicine, College of Medicine, National Taiwan University, Taipei, Taiwan

Correction to: *Scientific Reports* 10.1038/s41598-022-18992-1, published online 26 August 2022

In the original version of this Article, Sheng-Che Chou was incorrectly listed as the corresponding author. The correct corresponding author for this Article is Sheng-Hong Tseng. Correspondence and request for materials should be addressed to shenghongtseng@gmail.com.

The original Article has been corrected.

